# Molecular Ir-Based Coordination Compound Grafted onto Covalent Organic Framework for Efficient Photocatalytic H_2_ Evolution

**DOI:** 10.3390/ma18081874

**Published:** 2025-04-19

**Authors:** Chao Wu, Haoyan Zhang, Xuan Zheng, Jing Ding, Yuanyuan Li, Feiyong Chen, Zhengfeng Zhao

**Affiliations:** 1 Resources and Environment Innovation Institute, Shandong Jianzhu University, Jinan 250101, China; 13127158129@163.com; 2 School of Chemistry and Chemical Engineering, Qilu University of Technology (Shandong Academy of Sciences), Jinan 250353, China; y2463809770@163.com (H.Z.); zzf@qlu.edu.cn (Z.Z.); 3 Business School, Shandong Jianzhu University, Jinan 250101, China; 15688496290@163.com; 4 School of Energy and Machinery, Dezhou University, Dezhou 253023, China; dingjing@dzu.edu.cn

**Keywords:** covalent organic frameworks, molecular-level design, water splitting, hydrogen evolution, photocatalysis

## Abstract

The urgency of reducing pollution and developing clean energy storage requires efficient photocatalytic hydrogen evolution (PHE) tactics. To improve solar conversion efficiency, it is highly imperative to accelerate the photocarriers separation and transport through materials design. A stable hydrogen evolution photocatalyst based on TpPa-COFs (triformylphloroglucinol phenylenediamine covalent organic frameworks) was developed by a molecular-level design strategy. The study successfully introduced a molecular-scale Ir active site onto the surface of TpPa-COFs via coordination bonds. It verified the structural integrity of TpPa-COFs and the existence of Ir through the basic structural characterizations, such as Fourier transform infrared spectroscopy (FT-IR), X-ray photoelectron spectroscopy (XPS), and transmission electron microscopy (TEM). After the Ir-based coordination compound joining, the absorption edge of TpPa-COF-M1 and TpPa-COF-M2 was extended to 750 nm. The TpPa-COF + M1 exhibited the highest photocatalytic H_2_ evolution rate of 662 µmol/h (10 mg catalyst) under visible-light (λ ≥ 420 nm) irradiation. The apparent quantum yield (AQY) of TpPa-COF-M1 is calculated to be 1.9%, 3.8%, 4.8%, 2.8%, 1.8%, and 0.3% at monochromatic wavelengths of 420, 450, 470, 500, 550, and 600 nm, respectively. Our findings confirm that the molecular-level design of photocatalysts can effectively boost performance and reduce cost in photocatalytic reactions and provide an important strategy for designing efficient photocatalysts.

## 1. Introduction

The urgent need for clean and sustainable alternative energy sources accelerates the diversified development of new energy [1,2]. The use of solar photons to drive solar energy into energy storage has been energetically promoted as one of the most green and sustainable solutions in this regard [3,4]. The research of efficient photocatalytic water-splitting systems for hydrogen production is becoming an especially active field of energy research [5,6,7]. The advantages of strong, broad-spectrum absorption capability, rapid separation and transfer of photogenerated charges, and effective utilization of these photogenerated charges are necessary for an efficient photocatalyst [8,9]. Among these, the separation and effective utilization of photogenerated electrons have always been key problems in the field of photocatalysis [10]. Therefore, precisely controlling the photogenerated carrier motion path and effectively improving carrier separation efficiency are very important for optimizing the performance of photocatalysts [11]. Different scales of substances react with different material properties [12]. Designing materials at the atomic scale will produce better results than at the macroscopic scale [13].

**Figure 1:**
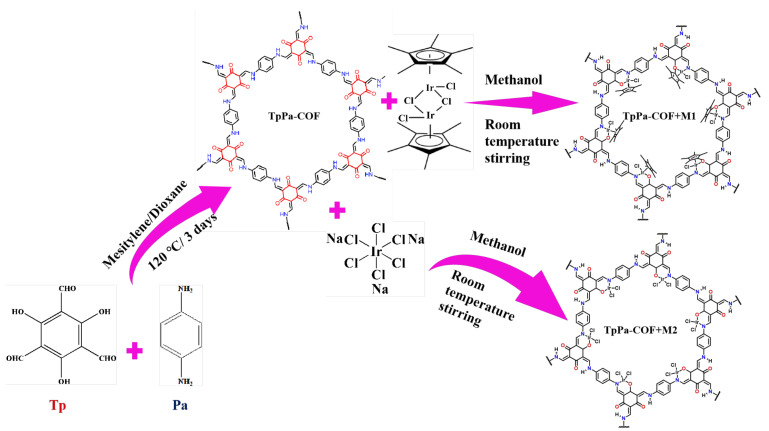
Synthesis and structure diagram of TpPa-COF, TpPa-COF + M1, and TpPa-COF + M2.

As a new class of photoactive materials for light-induced hydrogen evolution, covalent organic frameworks (COFs) have quickly gained popularity because of their efficient photocatalytic performance, controllable synthesis process, and stable chemical properties [14,15]. Even more so, COFs are adjustable for their (opto)electronic properties, structure, crystallinity, and porosity due to their modular, versatile, and adaptive construction, which provides great convenience for designing photocatalytic extrusion at the molecular level [16,17]. TpPa-COF is commonly used visible light photocatalytic for hydrogen production via water splitting [18,19]; its structure diagram is shown in Figure 1. TpPa-COFs have shown efficient photocatalytic hydrogen evolution performance and excellent visible light absorption ability with continuous improvement [18,20]. However, the high polarity imine and β-ketoenamine and the concomitantly discontinuous π-electron delocalization in TpPa-COFs are unfavorable for the migration of the photogenerated charge carriers in the host backbones [21]. Moreover, TpPa-COFs lack metal centers, which causes metal-containing cocatalysts to be required to achieve high photocatalytic efficiencies [22,23]. Therefore, the cocatalyst, which is not at the active site and easily clusters metal atoms, will cause the waste of precious metals [24,25]. It is of great significance to tailor-design steady and highly available hydrogen evolution active sites for hydrogen evolution reactions.

An effective strategy is to construct cocatalysts loaded structure directly connected to the active site with strong interfacial effects, which ensure directional transmission of the photogenerated electrons [26,27,28]. However, the development of such a system is challenging because efficient proton transport and reduction reactions require cocatalysts’ strong coupling of the light-harvesting and charge percolation processes on the COF [29,30]. Therefore, the choice of cocatalyst ligand is very important. Molecular metal complex catalysts have high tunability in water reduction performance due to their flexible molecular design. At present, molecular catalysts have been widely used in electrocatalysis, photocatalysis, and other fields, and show excellent results [31,32,33].

In this report, molecular catalysts were successfully introduced into the photocatalytic field as cocatalysts to improve the photocatalytic hydrogen evolution property of TpPa-COFs. Since most of the Ir-based coordination compound have good hydrogen evolution properties [34,35], two kinds of Ir-based coordination compound were selected as molecular catalysts for self-assembly with TpPa-COFs and the hydrogen production properties of the products were studied. The Ir-based coordination compound was combined with TpPa-COFs to prepare TpPa-COF + M1 and TpPa-COF + M2 using a self-assembly method (Figure 1). Efficient hydrogen evolution is seen with single-site linked Ir-based cocatalysts. The methodology can also be extended to other complex molecular catalysts and COFs. The results provide a pathway for the development of efficient and steady single-site heterogeneous photosynthesis systems that can precisely control the properties, density, and distribution of photocatalytic active sites.

## 2. Experimental

**Synthesis of TpPa-COF:** The TpPa-COF was synthesized using Schiff-base reactions. Triformylphloroglucinol (Tp, 63 mg, 0.3 mmol) (Zhongke, Jilin, China) and p-Phenylenediamine (Pa, 48 mg, 0.45 mmol) (Aladdin, Shanghai, China) were mixed together in the presence of aqueous acetic acid (0.5 mL, 3 mol/L) (Aladdin, Shanghai, China) using the mixed solution of mesitylene (1.5 mL) (Aladdin, Shanghai, China) and dioxane (1.5 mL) (Aladdin, Shanghai, China) as the solvent. After the mixture was sufficiently uniform, this mixture was flash frozen at 77 K (liquid N_2_ bath) and degassed in three freeze-pump-thaw cycles. Next, the mixture was heated at 120 °C for 3 days. The obtained powder was rinsed with methanol and dried under a vacuum to obtain TpPa-COF.

**Synthesis of TpPa-COF+M1:** 3.5 mg (0.004 mmol) pentamethylcyclopentadienyl iridium (Aladdin, Shanghai, China) was added into 10 mL methanol, which was named M1. Different quantities of M1 (1 mL, 2 mL, 3 mL, and 5 mL) were mixed with 10 mg of TpPa-COF powder. The mixture was well-dispersed by ultrasonication for 10 min and then stirred overnight. Finally, the mixture was repeatedly cleaned with methanol and natural drying. The samples were denoted as 1% M1-TpPa, 2% M1-TpPa, 3% M1-TpPa, and 5% M1-TpPa, respectively. The optimized specimens from the M1-series were systematically selected for comprehensive characterization based on their superior photocatalytic performance in hydrogen evolution reactions, which is uniformly represented by TpPa-COF + M1.

**Synthesis of TpPa-COF+M2:** 2 mg (0.004 mmol) iridium sodium chloride (Aladdin, Shanghai, China) was added into 5 mL methanol, which was named M2. Different quantities of M2 (1 mL and 2 mL) were mixed with 10 mg of TpPa-COF powder. The mixture was well-dispersed by ultrasonication for 10 min and then stirred overnight. Finally, the mixture was repeatedly cleaned with methanol and natural drying. The samples were denoted as 1% M2-TpPa and 2% M2-TpPa, respectively. The optimized specimens from the M2-series were systematically selected for comprehensive characterization based on their superior photocatalytic performance in hydrogen evolution reactions, which is uniformly represented by TpPa-COF + M2.

The experiment on hydrogen production by photocatalytic water splitting, methods of characterizing the materials, and the formula of apparent quantum yield (AQY) are described in detail in the Supporting Information.

**Abbreviation description:** M1 corresponds to pentamethylcyclopentadienyl iridium; M2 corresponds to iridium sodium chloride; TpPa-COF + M1 corresponds to 3% M1-TpPa; TpPa-COF + M2 corresponds to 1% M2-TpPa.

### 2.1. Photocatalytic Hydrogen Production

The photoelectrochemical measurements were conducted using a Gamry Interface 1000 electrochemical workstation (Gamry Instruments Inc., Warminster, PA, USA) equipped with a three-electrode configuration. The electrolyte consisted of 0.5 M Na_2_SO_4_ aqueous solution, where the synthesized catalyst sample, an Ag/AgCl electrode, and a platinum foil served as the working electrode, reference electrode, and counter electrode, respectively.

For the photocatalytic hydrogen evolution evaluation, experiments were performed in a quartz reactor integrated with a closed gas recirculation system and an external cooling circulator. A 300-W xenon arc lamp (PLSSXE300D/300DUV, Beijing Perfectlight, Beijing, China) served as the irradiation source, with a UV cutoff filter (λ > 420 nm) to eliminate ultraviolet components. Prior to testing, 10 mg of the catalyst was ultrasonically dispersed for 30 min in 100 mL of aqueous solution containing 0.02 M ascorbic acid as a sacrificial agent. The evolved gases were continuously analyzed using an online gas chromatograph (GC) equipped with a thermal conductivity detector (TCD) (Ruihong, Dezhou, China). Apparent quantum yield (AQY) measurements were performed under monochromatic light irradiation using wavelength-specific band-pass filters (420 nm, 450 nm, 480 nm, 500 nm, 550 nm, and 600 nm), with photon flux calibrated by a silicon photodiode.

### 2.2. Characterization

Crystalline phase analysis was performed using a Bruker D8 Advance X-ray diffractometer (XRD) (Bruker, Billerica, MA, USA) with Cu Kα radiation (λ = 0.154178 nm), operating at 40 kV and 40 mA. Morphological characterization was conducted via transmission electron microscopy (TEM, JEOL-2100, JEOL, Tokyo, Japan) at an accelerating voltage of 200 kV. Chemical functional groups were identified by Fourier transform infrared spectroscopy (FT-IR, Bruker TENSOR II, Bruker, Billerica, MA, USA) using KBr-pressed pellets (Aladdin, Shanghai, China), with spectra recorded in the range of 4000–500 cm^−1^ at ambient conditions. Elemental composition was quantified through inductively coupled plasma optical emission spectrometry (ICP-OES, PerkinElmer Avio 500, PerkinElmer, Waltham, MA, USA) with axial plasma viewing, calibrated using certified multi-element standard solutions. Structural disorder analysis was performed via Raman spectroscopy (Horiba LabRAM HR Evolution, HORIBA, Kyoto, Japan) using a 532 nm diode laser excitation source (5 mW power) with 1800 grooves/mm grating, acquiring spectra in the 100–2000 cm^−1^ range. Surface properties were evaluated through nitrogen adsorption-desorption isotherms at 77 K using a BEL Sorp-II mini analyzer (BSD Instrument, Beijing, China) for Brunauer-Emmett-Teller (BET) surface area and pore size distribution calculations. Optical absorption characteristics were determined by diffuse reflectance spectroscopy (DRS) on a Shimadzu UV-2700 spectrophotometer (Shimadzu, Kyoto, Japan) equipped with an integrating sphere, using BaSO_4_ as the reflectance standard. Photoluminescence (PL) emission spectra were acquired at room temperature using a Hitachi F-4600 fluorescence spectrophotometer (Hitachi, Tokyo, Japan) with a 325 nm xenon lamp excitation source. Surface chemical states were analyzed by X-ray photoelectron spectroscopy (XPS) on a PHI5000 VersaProbe III (Physical Electronics, Chanhassen, MN, USA) system with monochromatic Al Kα radiation (1486.6 eV), and binding energies were calibrated against the C 1s peak at 284.8 eV.

## 3. Results and Discussion

**Figure 2:**
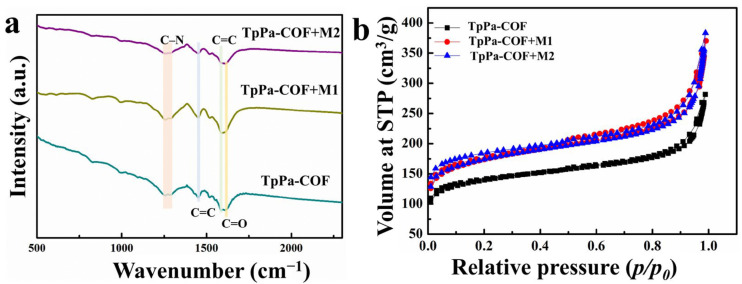
(**a**) Comparison of the FT-IR spectra of TpPa-COF, TpPa-COF + M1, and TpPa-COF + M2; (**b**) N_2_ adsorption/desorption isotherms of TpPa-COF, TpPa-COF + M1, and TpPa-COF + M2.

The structure of TpPa-COF was verified by powder X-ray diffraction (PXRD), with the corresponding pattern shown in Figure S1. The (100) plane of TpPa-COF is proven to exist by the intense peak at ~4.7°. The peak at ~26.7° arises from π–π stacking interactions and corresponds to the (001) plane of TpPa-COF [36]. The experimental PXRD patterns of TpPa-COF match well with the simulated ones, confirming the successful synthesis of a highly crystalline framework. Figure 2a shows the FT-IR spectrum of TpPa-COF, TpPa-COF + M1, and TpPa-COF + M2. The FT-IR spectra in TpPa-COF match well with the compound, which exists as a β-ketoenamine-linked form [37]. The C–N stretching in TpPa-COF appears at 1250 cm^−1^ and 1284 cm^−1^. The peaks at 1444 cm^−1^ and 1578 cm^−1^ are derived from the aromatic C=C and C=C stretching bands, respectively [36,38,39]. The peak at 1616 cm^−1^ corresponds to the C=O stretching band. The FT-IR spectra in TpPa-COF + M1 and TpPa-COF + M2 are similar to the FT-IR spectra of TpPa-COF. There is no signal for connection between TpPa-COF and Ir-based coordination compound, which is because of the minimal amount of the Ir-based coordination compound. The specific surface areas of the TpPa-COF, TpPa-COF + M1, and TpPa-COF + M2 were investigated. Figure 2b shows the nitrogen adsorption-desorption isothermal curve. The specific surface areas of TpPa-COF, TpPa-COF + M1, and TpPa-COF + M2 were measured to be 442, 552, and 553 m^2^/g, respectively. The insignificant change between TpPa-COF + M1 and TpPa-COF + M2 indicates that the introduction of an Ir-based coordination compound does not change the specific surface area of the sample, because the concentration of Ir-based coordination compound in the sample is little or no more than 1%. Compared with TpPa-COF, the specific surface area of TpPa-COF + M1 and TpPa-COF + M2 increased slightly, because TpPa-COF + M1 and TpPa-COF + M2 underwent overnight stirring in methanol, which resulted in the sample stripping.

**Figure 3:**
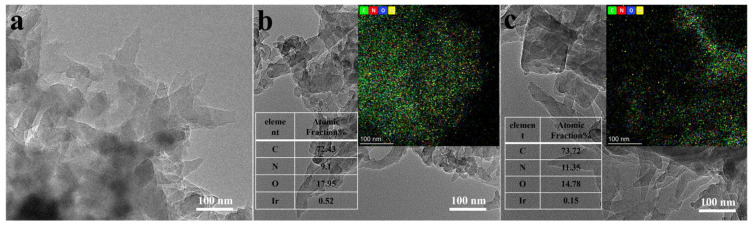
TEM images: (**a**) TpPa-COFl; (**b**) TpPa-COF + M1; (**c**) TpPa-COF + M2.

The transmission electron microscope (TEM) image demonstrates that TpPa-COF is composed of rod-like nanostructures (Figure 3a), which is the conventional morphology of compound TpPa-COF. As shown in Figure 3b,c, TpPa-COF + M1 and TpPa-COF + M2 exhibit a similar pattern to TpPa-COF. No Ir-based coordination compound can be seen in TpPa-COF + M1 and TpPa-COF + M2 because the amount of Ir-based coordination compound is very small. As can be seen from the picture of mapping, the element Ir does exist, but the atomic content of Ir in TpPa-COF + M1 and TpPa-COF + M2 is only 0.52% and 0.15%, respectively. Therefore, it is difficult to find Ir-based coordination compounds in TEM images.

**Figure 4:**
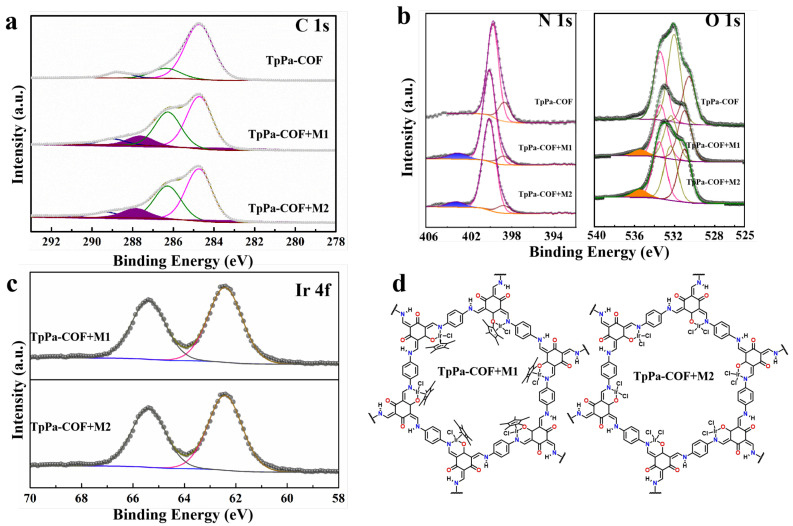
XPS spectra of TpPa-COF, TpPa-COF + M1, and TpPa-COF + M2: (**a**) C 1s; (**b**) N 1s and O 1s; (**c**) Ir 4f; (**d**) coordination diagram.

To further test the existence of element Ir and analyze the element valence, X-ray photoelectron spectroscopy (XPS) tests have been used (Figure 4). In high-resolution spectra, the C 1s spectrum of TpPa-COF displays three main peaks at 284.8, 286.2, and 288.6 corresponding to C=C, C–N, and C=O, respectively (Figure 4a) [40]. The N 1s peaks of TpPa-COF correspond to C=N (398.6 eV) and C–N (399.8 eV) (Figure 4b). The deconvoluted O 1s XPS spectra of TpPa-COF provide three main peaks at 530.5, 532.0, and 533.4 eV, corresponding to H_2_O, C=O, and adsorption O_2_, respectively (Figure 4b) [41]. These prove that the chemical structure of TpPa-COF is complete. After M1 and M2 Ir-based coordination compound joining, all peaks in C 1s, N 1s, and O 1s orbital have obvious deviation compared with TpPa-COF, which suggests that there is a charge transfer between TpPa-COF and the Ir-based coordination compound. The appearance of C-O (around 287.8 eV) in TpPa-COF + M1 and TpPa-COF + M2 C 1s XPS spectra is due to the destruction of C=O by the Ir-based compound. A new N signal can be observed on TpPa-COF + M1 and TpPa-COF + M2 N 1s XPS spectra around 403 eV, assigned to the Ir-N coordination bond [42]. Meanwhile, the new O signal on TpPa-COF+M1 and TpPa-COF + M2 O 1s XPS spectra around 535 eV are assigned to the Ir-O coordination bond. These signals prove that both M1 and M2 Ir-based coordination compounds were successfully coordinated with TpPa (Figure 4d). The high-resolution Ir 4f shows two peaks located around 62.2 and 65.5 eV indexed to Ir (IV) species (Figure 4c) [43]. As for TpPa-COF+M1, the valence state of Ir changes from 3^+^ to 4^+^ after combining with TpPa-COF, which further proves that the transport of electrons has taken place between the M1 Ir-based coordination compound and TpPa-COF. The valence state of Ir in TpPa-COF + M2 does not change, which indicates that TpPa-COF stabilizes the M2 Ir-based coordination compound by sharing electron pairs.

**Figure 5:**
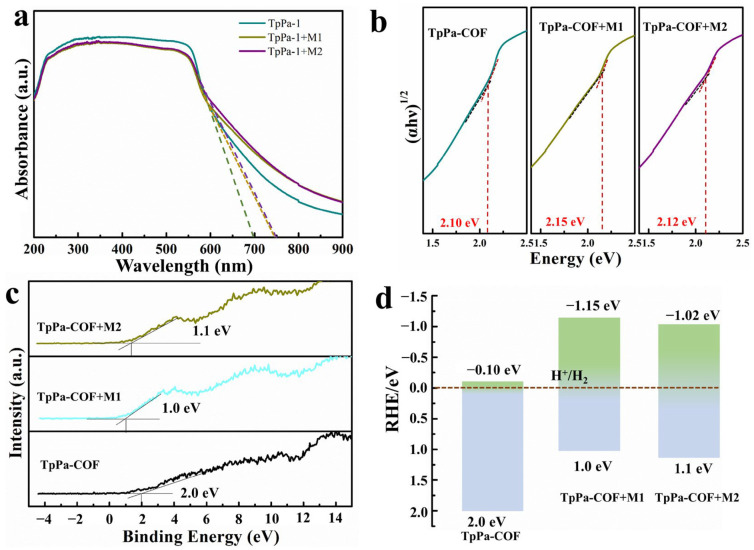
The UV–Vis DRS (**a**), the optical bandgaps (**b**), XPS Valence-band spectrum (**c**) and the calculated positions of LUMO and HOMO (**d**) of TpPa-COF, TpPa-COF + M1, and TpPa-COF + M2.

UV-vis diffuse reflectance spectroscopy (UV-DRS) and XPS valence band spectra were employed to determine the band structures of the samples (Figure 5). As shown in Figure 5a, the obvious visible light absorbance edge was exhibited in TpPa-COF, around 700 nm, which is consistent with reported data [44], while the absorbance edge was red-shifted to around 750 nm for TpPa-COF + M1 and TpPa-COF + M2. This result shows that the absorption edge of TpPa-COF is extended when an Ir-based coordination compound is added and the type of Ir-based coordination compound had little effect on the absorbance edge. Furthermore, the corresponding band gap of TpPa-COF, TpPa-COF + M1, and TpPa-COF + M2 are calculated to be 2.10, 2.15, and 2.12 eV, respectively, according to the method provided in the literature [45] (Figure 5b). These results prove that the Ir-based coordination compound can widen the light absorption edge and reduce the band gap of the photocatalyst. To further investigate the semiconductor characteristics, the XPS valence band spectrum was measured for TpPa-COF, TpPa-COF + M1, and TpPa-COF + M2 (Figure 5c). The E_VB_ of TpPa-COF was determined to be 2.0 eV. Compared to the TpPa-COF, the E_VB_ of TpPa-COF + M1 and TpPa-COF + M2 shifts to 1.0 eV and 1.1 eV. The position of LUMO of TpPa-COF, TpPa-COF + M1, and TpPa-COF + M2 were obtained by combining these measurements with the values of band gap, which can be flagged as −0.10 eV, −1.15 eV, and −1.02 eV, respectively (Figure 5d). The LUMO position of TpPa-COF, TpPa-COF + M1, and TpPa-COF + M2 above the relative hydrogen electrode indicate that they both possess the ability to reduce water. The most negative LUMO position of TpPa-COF + M1 corresponds to the highest electron reduction capacity.

**Figure 6:**
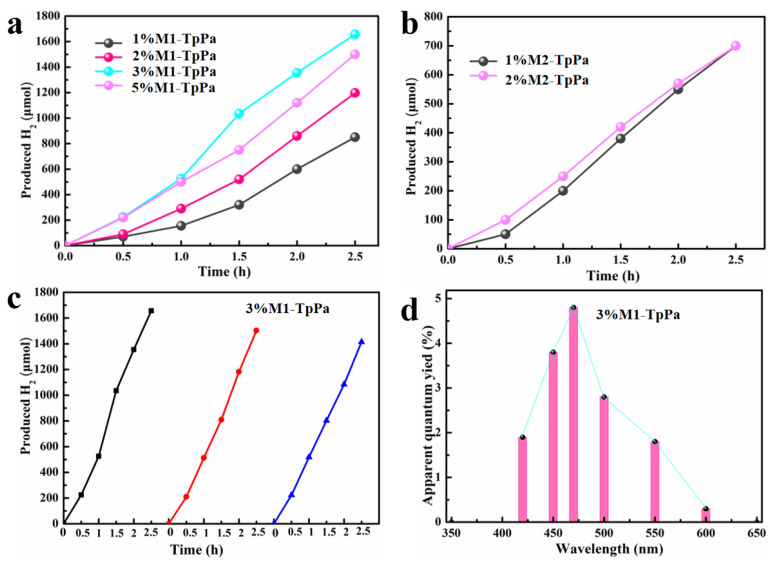
The photocatalytic properties of H_2_ production under visible light of 1% M1-TpPa, 2% M1-TpPa, 3% M1-TpPa, and 5% M1-TpPa (**a**), the photocatalytic properties of H_2_ production under visible light of 1% M2-TpPa and 2% M2-TpPa (**b**), the recyclability of H_2_ production of 3% M1-TpPa under visible light (**c**), The AQY of 3% M1-TpPa under different wavelengths (**d**).

The photocatalytic hydrogen evolution performance of TpPa-COF, TpPa-COF + M1, and TpPa-COF + M2 was evaluated under visible light using ascorbic acid sacrificial reagent. As shown in Figure S2, the dosage of catalyst was determined to be 10 mg after quality optimization, although it showed ~5% higher activity when the dosage was 20 mg. The loading amount of Ir-based coordination compound is closely related to the photocatalytic activity (Figure 6a). According to the results of ICP (Table S1), the actual mass percentage of Ir atoms in the 1% M1-TpPa, 2% M1-TpPa, 3% M1-TpPa, 5% M1-TpPa, 1% M2-TpPa, and 2% M2-TpPa are 0.70%, 1.66%, 2.51%, 4.24%, 0.62%, and 1.11%, respectively. 3% M1-TpPa provides the highest hydrogen evolution rate of 662 µmol/h. The pristine TpPa-COFs without loading of Ir-based coordination compound have no photocatalytic activity, validating that the Ir-based coordination compound provides catalytic active sites for hydrogen generation. 1% M1-TpPa and 2% M1-TpPa, with less Ir-based coordination compound, could not provide enough photocatalytic active sites, affording hydrogen evolution rates of 155 µmol/h and 290 µmol/h, respectively. The excessive amounts of Ir-based coordination compound in 5% M1-TpPa probably inhibit the light absorption process and create an excess charge recombination center, leading to the declined hydrogen evolution rates of 600 µmol/h. TpPa-COF+M2 with 1% and 2% loading amount of Ir-based coordination compound provides a similar hydrogen evolution rate of 250 µmol/h (Figure 6b), which is significantly lower than TpPa-COF + M1. This is because the Ir^4+^ in M2 is reduced under light, which can be confirmed in Figure S3. As Figure S3 shows, the surface of TpPa-COF + M1 is still smooth after the photocatalytic reaction, but many nanoparticles can be observed on the surface of TpPa-COF + M2, which indicates that Ir^4+^ in M2 aggregates to large particles under illumination, resulting in lower performance. From this, it can be seen that organic ligands (pentamethylcyclopentadienyl) stabilize Ir atoms more easily than inorganic ligands (sodium chloride). To further verify the stability of TpPa-COF + M1 during the photocatalytic hydrogen evolution test, pre- and post-reaction FT-IR and Raman tests were provided, as shown in Figure S5. Compared with the TpPa-COF + M1 before the reaction, the FT-IR and Raman absorption peaks of the TpPa-COF+M1 after the reaction did not shift and no new diffraction peak appeared, which proved that the chemical structure of TpPa-COF + M1 before and after the photocatalytic hydrogen evolution test was stable. And the difference between the mass percentage of TpPa-COF + M1 before (2.51%) and after (2.35%) the photocatalytic hydrogen evolution test is very small, which proves that Ir are not consumed during the test (Table S1). Atom Ir loaded onto the surface of TpPa-COF by light deposition (3% Ir-TpPa, 100 µmol/h) exhibits significantly lower photocatalytic efficiency than Ir loaded by molecular coordination (TpPa-COF + M1/TpPa-COF + M2), which indicates that loading cocatalyst in the form of molecular coordination can effectively improve the photocatalytic activity (Figure S4a). The clumped atomic particles observed in the TEM image of Ir cocatalyst in atomic form after reaction also confirmed the waste of metal (Figure S4b). Meanwhile, the activity of the TpPa-COF + M1 is also higher than that of 3% Rh/TpPa-COF (540 µmol/h), indicating that M1 Ir-based coordination compounds could serve as promising alternatives to rhodium and other precious metals as cocatalysts to effectively enhance photocatalytic hydrogen evolution activity of COFs semiconductors (Figure S4a).

TpPa-COF + M1 shows excellent durability and photochemical stability; there are no detectable activity loss and structural variation after 3 cycles for photocatalytic hydrogen evolution reaction (Figure 6c). The light-wavelength dependency examination shows that TpPa-COF + M1 could use a broad range of visible light, and hydrogen production rates under monochromatic light of 420, 450, 470, 500, 550, and 600 nm are 6.5, 18, 24.72, 25.41, 10.02, and 1.68 µmol h^−1^, respectively (Figure 6d). The apparent quantum yields (AQY) are calculated to be 1.9%, 3.8%, 4.8%, 2.8%, 1.8%, and 0.3%, respectively.

**Figure 7:**
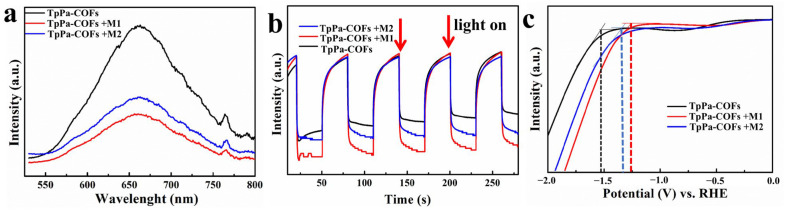
The Photoluminescence of TpPa-COF, TpPa-COF + M1, and TpPa-COF + M2 (**a**), transient photocurrent response curves of TpPa-COF, TpPa-COF + M1, and TpPa-COF + M2 (**b**), the HER (Hydrogen Evolution Reaction) polarization curves of TpPa-COF, TpPa-COF + M1, and TpPa-COF + M2 (**c**).

Photogenerated charge separation was tested for the mechanism of photocatalytic performance enhancement. As shown in Figure 7a, the steady-state photoluminescence (PL) spectra were performed with excitation at 380 nm. Compared to TpPa-COF, TpPa-COF+M1 and TpPa-COF+M2 exhibited obvious PL quenching at the emission peak of 640 nm, which indicates that the Ir-based coordination compound can effectively inhibit the recombination of the photogenerated electron and hole. The lowest PL peak intensity of TpPa-COF + M1 indicates that the formation of coordination bond between TpPa-COF and Ir-based coordination compound is more conducive to electron transport. The photocurrent density is used to measure the response of the catalyst to photoelectricity (Figure 7b). The photocurrent density of TpPa-COF + M1 and TpPa-COF + M2 is remarkably increased when compared with that of TpPa-COF. Moreover, TpPa-COF + M1 shows the most significant photoelectric response in four cycles, which indicates that TpPa-COF + M1 can produce more effective electrons. The conduction of photogenerated electrons through Ir-based coordination compound molecules is crucial for enhancing photocatalytic performance. Therefore, electrochemical methods were used to investigate the electron conduction of Ir-based coordination compound molecules. It can be observed that the trend of hydrogen evolution overpotentials was TpPa-COF + M1 < TpPa-COF + M2 < TpPa-COFs in linear sweep voltammetry (LSV) (Figure 7c). A maximum overpotential on the TpPa-COFs electrode implied that the photoelectrons of TpPa-COFs are difficult to participate in the reduction reaction of water. After the introduction of the Ir-based coordination compound, the overpotential decreased significantly, which indicates Ir-based coordination compound contributes to the adsorption and dissociation of H^+^ [46]. The lowest overpotential of TpPa-COF + M1 indicates that it needs to overcome the least resistance in the water-splitting process. These results all indicate that TpPa-COF + M1 has a superior water-splitting capacity, corresponding to the hydrogen production results, which can be attributed to the M1 Ir-based coordination compound co-optimizing the light absorption capacity, the photogenerated carrier transfer and separation efficiency, and the surface reactive active site [47,48].

**Figure 8:**
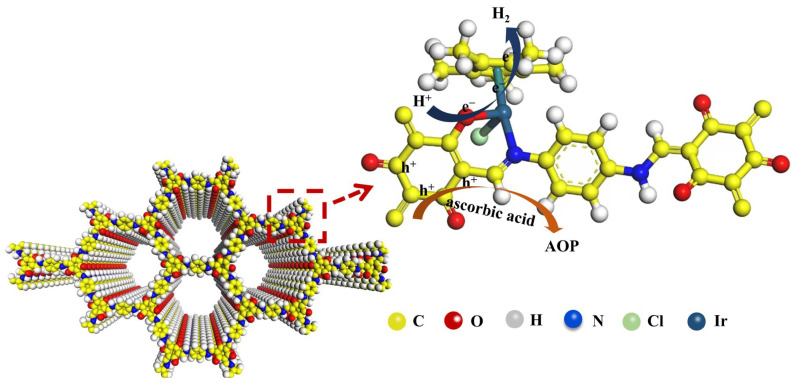
The electron transfer mechanism in TpPa-COF + M1 photocatalyst.

As shown in the molecular diagram in Figure 8, TpPa-COF + M1 was prepared by a halogenating reaction between TpPa-COF and the M1 Ir-based coordination compound. This covalent immobilization can resolve the leaching issues of catalysts, particularly for ionic molecules. The combination of iridium complexes and TpPa-COFs changes the way electrons are transported in TpPa-COFs. When visible light illuminates TpPa-COFs, the electron holes in TpPa-COFs are excited. Due to the higher water-splitting potential of traditional TpPa-COFs, electrons cannot fully participate in the decomposition water reaction after being excited, and finally recombine with the hole. Therefore, although traditional TpPa-COFs have a strong visible light response, they show almost no photocatalytic hydrogen evolution performance. When Ir-based coordination compounds are added, the strong conductivity of the iridium atom rapidly attracts the excited electrons in TpPa-COFs, in which the electron transfer process is faster than the electron-hole recombination between the VB and CB of TpPa-COFs. At the same time, the iridium atom can reduce the activation energy of water and promote the water-splitting reaction. In this case, the lifetime of photogenerated electrons at TpPa-COFs becomes longer and electron utilization increases significantly. Finally, the performance of hydrogen production by photocatalytic water splitting is obviously improved. Comparative analysis with previously reported systems (Table S2) demonstrates that precisely controlled grafting of a cocatalyst via molecular coordination not only enhances photocatalytic activity through optimized charge separation, but also reduces precious metal consumption while maintaining stability, which provides a new idea for designing efficient and cheap water-splitting photocatalysts.

## 4. Conclusions

In summary, an Ir-molecular-complex-modified TpPa-COF photocatalyst was successfully fabricated. The TpPa-COF + M1 photocatalyst demonstrates a remarkable photocatalytic hydrogen evolution rate of 662 µmol h^−1^ and an apparent quantum yield (AQY) of 4.8% at 470 nm. This study reveals that both charge separation efficiency and light absorption are significantly enhanced through the introduction of the Ir complex into the TpPa-COF framework. A highly active Ir molecular complex cocatalyst was covalently anchored to TpPa-COF, effectively reducing the hydrogen evolution overpotential in water splitting. The photocatalytic performance was dramatically enhanced via coordination bonding with molecular cocatalysts. Organic ligands exhibit superior stability in preserving their structural and electronic properties compared to inorganic ligands. This study provides a facile strategy to modulate the electronic structure of TpPa-COF and proposes a novel approach for designing molecular-complex-based cocatalysts. These findings offer critical insights into advancing solar-to-hydrogen conversion technologies.

## Data Availability

The original contributions presented in this study are included in the article/Supplementary Materials. Further inquiries can be directed to the corresponding authors.

## Supplementary Materials

The following supporting information can be downloaded at: https://www.mdpi.com/article/10.3390/ma18081874/s1, Figure S1: Powder X-ray diffraction (PXRD) analysis of TpPa-COFs; Figure S2: Photocatalytic hydrogen evolution performance of different amounts of catalyst under visible light irradiation; Figure S3: TEM images after the reaction: (a) TpPa-COF + M1; (b) TpPa-COF + M2; Figure S4: (a): Photocatalytic hydrogen evolution performance of TpPa-COFs with different kinds of molecules ligands under visible light irradiation. 3% Ru-TpPa means that RhCl2 with 3% mass percentage deposited onto TpPa COFs by photodeposition. 3% Ir-TpPa means that C_20_H_30_Cl_4_Ir_2_ ((Pentamethylcyclopentadienyl)iridium(III) chloride dimer) with 3% mass percentage deposited onto TpPa COFs by photodeposition. (b) The TEM and HRTEM images of 3% Ir-TpPa after Photocatalytic hydrogen evolution test; Figure S5: The absorption peaks of TpPa-COFs + M1 before and after photocatalytic hydrogen evolution tests: (a) Raman; (b) FTIR; Table S1: Ir Mass percentage of different samples tested by ICP; Table S2: Comparison of typical TpPa-COF photocatalysts reported for the hydrogen production efficiency [18,37,41,49,50,51,52,53,54].

## References

[1] Klingler S., Bagemihl B., Mengele A.K., Kaufhold S., Myllyperkiö P., Ahokas J., Pettersson M., Rau S., Mizaikoff B. (2023). Rationalizing In Situ Active Repair in Hydrogen Evolution Photocatalysis via Non-Invasive Raman Spectroscopy. Angew. Chem. Int. Ed..

[2] Silver S.C., Niklas J., Du P., Poluektov O.G., Tiede D.M., Utschig L.M. (2013). Protein Delivery of a Ni Catalyst to Photosystem I for Light-Driven Hydrogen Production. J. Am. Chem. Soc..

[3] Sun K., Qian Y., Li D., Jiang H.L. (2024). Reticular Materials for Photocatalysis. Adv. Mater..

[4] Liu J., Yu Y., Qi R., Cao C., Liu X., Zheng Y., Song W. (2019). Enhanced electron separation on in-plane benzene-ring doped g-C_3_N_4_ nanosheets for visible light photocatalytic hydrogen evolution. Appl. Catal. B Environ..

[5] Abhishek B., Jayarama A., Rao A.S., Nagarkar S.S., Dutta A., Duttagupta S.P., Prabhu S.S., Pinto R. (2024). Challenges in photocatalytic hydrogen evolution: Importance of photocatalysts and photocatalytic reactors. Int. J. Hydrogen Energy.

[6] Yu W., Hu C., Bai L., Tian N., Zhang Y., Huang H. (2022). Photocatalytic hydrogen peroxide evolution: What is the most effective strategy?. Nano Energy.

[7] Chang Y.-C., Bi J.-N., Pan K.-Y., Chiao Y.-C. (2024). Microwave-Assisted Synthesis of SnO_2_@ZnIn_2_S_4_ Composites for Highly Efficient Photocatalytic Hydrogen Evolution. Materials.

[8] Dai C., Liu B. (2020). Conjugated polymers for visible-light-driven photocatalysis. Energy Environ. Sci..

[9] Fang Y., Hou Y., Fu X., Wang X. (2022). Semiconducting Polymers for Oxygen Evolution Reaction under Light Illumination. Chem. Rev..

[10] Sun L., Han L., Huang J., Luo X., Li X. (2022). Single-atom catalysts for photocatalytic hydrogen evolution: A review. Int. J. Hydrogen Energy.

[11] Chu X., Sathish C.I., Yang J.H., Guan X., Zhang X., Qiao L., Domen K., Wang S., Vinu A., Yi J. (2023). Strategies for Improving the Photocatalytic Hydrogen Evolution Reaction of Carbon Nitride-Based Catalysts. Small.

[12] Liu Q., Wang X. (2022). Sub-nanometric materials: Electron transfer, delocalization, and beyond. Chem Catal..

[13] Cheng X., Zhang S., Wang X. (2021). Cluster–Nuclei Coassembled One-Dimensional Subnanometer Heteronanostructures. Nano Lett..

[14] Wang L., Zhang Y. (2024). Impact of Interfaces on the Performance of Covalent Organic Frameworks for Photocatalytic Hydrogen Production. Small.

[15] Yang F., Qu J., Zheng Y., Cai Y., Yang X., Li C.M., Hu J. (2022). Recent advances in high-crystalline conjugated organic polymeric materials for photocatalytic CO_2_ conversion. Nanoscale.

[16] Wu Y., Wang R., Kim Y. (2024). Single-Atom Catalysts on Covalent Organic Frameworks for Energy Applications. ACS Appl. Mater. Interfaces.

[17] Li Y., Song X., Zhang G., Wang L., Liu Y., Chen W., Chen L. (2022). 2D Covalent Organic Frameworks Toward Efficient Photocatalytic Hydrogen Evolution. ChemSusChem.

[18] Jiang S., Niu H., Sun Q., Zhao R., Li N., Cai Y. (2024). Significant improvement of photocatalytic hydrogen evolution performance in covalent organic frameworks: Substituent fine-tuning. J. Mater. Chem. A.

[19] Wang S.-D., Huang L.-Y., Xue L.-J., Kang Q., Wen L.-L., Lv K.-L. (2024). Sulfur-vacancy-modified ZnIn_2_S_4_/TpPa-1 S-scheme heterojunction with enhanced internal electric field for boosted photocatalytic hydrogen production. Appl. Catal. B Environ. Energy.

[20] Yang D., Li Z.-G., Zhang X., Ren Z., Lu W., Liu H., Guo X., Zhang J., Bu X.-H. (2023). Rational design of ZnCdS/TpPa-1-COF heterostructure photocatalyst by strengthening the interface connection in solar hydrogen production reactions. Nano Res..

[21] Yin L., Zhao Y., Xing Y., Tan H., Lang Z., Ho W., Wang Y., Li Y. (2021). Structure-Property relationship in β-keto-enamine-based covalent organic frameworks for highly efficient photocatalytic hydrogen production. Chem. Eng. J..

[22] Yan G., Sun X., Zhang K., Zhang Y., Li H., Dou Y., Yuan D., Huang H., Jia B., Li H. (2022). Integrating Covalent Organic Framework with Transition Metal Phosphide for Noble-Metal-Free Visible-Light-Driven Photocatalytic H_2_ Evolution. Small.

[23] Dong P., Wang Y., Zhang A., Cheng T., Xi X., Zhang J. (2021). Platinum Single Atoms Anchored on a Covalent Organic Framework: Boosting Active Sites for Photocatalytic Hydrogen Evolution. ACS Catal..

[24] Shao M., Chen H., Hao S., Liu H., Cao Y., Zhao Y., Jin J., Dang H., Meng Y., Huo Y. (2022). N-doped vanadium carbide combined with Pt as a multifunctional cocatalyst to boost photocatalytic hydrogen production. Appl. Surf. Sci..

[25] Yao Y.-H., Yang Y., Wang Y., Zhang H., Tang H.-L., Zhang H.-Y., Zhang G., Wang Y., Zhang F.-M., Yan H. (2022). Photo-induced synthesis of ternary Pt/rGO/COF photocatalyst with Pt nanoparticles precisely anchored on rGO for efficient visible-light-driven H2 evolution. J. Colloid Interface Sci..

[26] Wang Y., Qu Y., Qu B., Bai L., Liu Y., Yang Z.D., Zhang W., Jing L., Fu H. (2021). Construction of Six-Oxygen-Coordinated Single Ni Sites on g-C_3_N_4_ with Boron-Oxo Species for Photocatalytic Water-Activation-Induced CO_2_ Reduction. Adv. Mater..

[27] Lazaar N., Wu S., Qin S., Hamrouni A., Bikash Sarma B., Doronkin D.E., Denisov N., Lachheb H., Schmuki P. (2025). Single-Atom Catalysts on C_3_N_4_: Minimizing Single Atom Pt Loading for Maximized Photocatalytic Hydrogen Production Efficiency. Angew. Chem. Int. Ed..

[28] Xiao X., Gao Y., Zhang L., Zhang J., Zhang Q., Li Q., Bao H., Zhou J., Miao S., Chen N. (2020). A Promoted Charge Separation/Transfer System from Cu Single Atoms and C_3_N_4_ Layers for Efficient Photocatalysis. Adv. Mater..

[29] Qiu Z., Luo Z., Zhou T., Zi B., Chen M., Hu R., Lv T., He T., Ma Y., Zhang J. (2025). Oxygen vacancy enriched and Cu single-atom contained covalent organic frameworks: A competitive photocatalyst to promote hydrogen evolution under visible light. Mater. Today Energy.

[30] Fang K., Chen Z., Wei Y., Fang S., Dong Z., Zhang Y., Li W., Wang L. (2022). Single site Co-S anchored on carbon nitride as a highly active cocatalyst for photocatalytic hydrogen evolution. J. Alloys Compd..

[31] Kumar R., Kumar S., Kailath A.J., Sahu R.K. (2024). Mechanistic investigation of hydrogen generation from water and magnesium catalyst reaction: Advanced reactive molecular dynamics simulation. Int. J. Hydrogen Energy.

[32] Messori A., Martelli G., Piazzi A., Basile F., De Maron J., Fasolini A., Mazzoni R. (2023). Molecular Ruthenium Cyclopentadienone Bifunctional Catalysts for the Conversion of Sugar Platforms to Hydrogen. ChemPlusChem.

[33] Li C.-B., Chu Y., Xie P., Xiong L., Wang N., Wang H., Tang J. (2020). Molecular Cobalt Catalysts Grafted onto Polymers for Efficient Hydrogen Generation Cathodes. Sol. RRL.

[34] Bodedla G.B., Tritton D.N., Chen X., Zhao J., Guo Z., Cham-Fai Leung K., Wong W.-Y., Zhu X. (2021). Correction to Cocatalyst-free Photocatalytic Hydrogen Evolution with Simple Heteroleptic Iridium(III) Complexes. ACS Appl. Energy Mater..

[35] Yao X., Fan L., Zhang Q., Zheng C., Yang X., Lu Y., Jiang Y. (2024). Impact of Anchoring Groups on the Photocatalytic Performance of Iridium(III) Complexes and Their Toxicological Analysis. Molecules.

[36] Kandambeth S., Mallick A., Lukose B., Mane M.V., Heine T., Banerjee R. (2012). Construction of Crystalline 2D Covalent Organic Frameworks with Remarkable Chemical (Acid/Base) Stability via a Combined Reversible and Irreversible Route. J. Am. Chem. Soc..

[37] Sheng J.L., Dong H., Meng X.B., Tang H.L., Yao Y.H., Liu D.Q., Bai L.L., Zhang F.M., Wei J.Z., Sun X.J. (2019). Effect of Different Functional Groups on Photocatalytic Hydrogen Evolution in Covalent-Organic Frameworks. ChemCatChem.

[38] Li C., Liu J., Li H., Wu K., Wang J., Yang Q. (2022). Covalent organic frameworks with high quantum efficiency in sacrificial photocatalytic hydrogen evolution. Nat. Commun..

[39] Ming J., Liu A., Zhao J., Zhang P., Huang H., Lin H., Xu Z., Zhang X., Wang X., Hofkens J. (2019). Hot pi-Electron Tunneling of Metal-Insulator-COF Nanostructures for Efficient Hydrogen Production. Angew. Chem. Int. Ed. Engl..

[40] He W., Kong K., Wang M., Dong B., Yuan D., Bryliakov K.P., Wang R. (2024). Photoelectron migration monitored by 3D orbital electron configuration of spinel cocatalysts for covalent organic framework-based photocatalytic hydrogen evolution. Appl. Catal. B Environ. Energy.

[41] Zhao Z., Chen W., Zhang G., Chen Y. (2023). Interface molecular wires induce electron transfer from COFs to Pt for enhanced photocatalytic H2 evolution. J. Mater. Chem. A.

[42] Zhao Z., Zheng D., Guo M., Yu J., Zhang S., Zhang Z., Chen Y. (2022). Engineering Olefin-Linked Covalent Organic Frameworks for Photoenzymatic Reduction of CO_2_. Angew. Chem. Int. Ed. Engl..

[43] Ding J., Li Z., Wang Y., Liu Y., Li F., Yu X., Huang P., Wang Y. (2025). Ir doping improved oxygen activation of WO_3_ for boosting acetone sensing performance at low working temperature. Appl. Surf. Sci..

[44] Yan M., Jiang F., Wu Y. (2023). Metal-free 2D-2D black phosphorus/covalent organic framework p-n heterojunction for efficient visible-light-driven hydrogen evolution without cocatalysts. Int. J. Hydrogen Energy.

[45] Patrycja M., Michał P., Wojciech M. (2018). How To Correctly Determine the Band Gap Energy of Modified Semiconductor Photocatalysts Based on UV−Vis Spectra. J. Phys. Chem. Lett..

[46] Wang Y., Huang Y., Liu S., Cui S., Zhang Y., Deng P. (2024). Iridium complex modified MOFs for enhancing photocatalytic hydrogen evolution. Energy Adv..

[47] Matt B., Fize J., Moussa J., Amouri H., Pereira A., Artero V., Izzet G., Proust A. (2013). Charge Photo-Accumulation and Photocatalytic Hydrogen Evolution Under Visible Light at an Iridium(III)-Photosensitized Polyoxotungstate. Energy Environ. Sci..

[48] Tritton D.N., Tang F.-K., Bodedla G.B., Lee F.-W., Kwan C.-S., Leung K.C.-F., Zhu X., Wong W.-Y. (2022). Development and advancement of iridium(III)-based complexes for photocatalytic hydrogen evolution. Coord. Chem. Rev..

[49] Li C.-C., Gao M.-Y., Sun X.-J., Tang H.-L., Dong H., Zhang F.-M. (2020). Rational combination of covalent-organic framework and nano TiO_2_ by covalent bonds to realize dramatically enhanced photocatalytic activity. Appl. Catal. B Environ..

[50] Chen Y., Yang D., Gao Y., Li R., An K., Wang W., Zhao Z., Xin X., Ren H., Jiang Z. (2021). On-Surface Bottom-Up Construction of COF Nanoshells towards Photocatalytic H_2_ Production. Research.

[51] Zhang L., Lu X., Sun J., Wang C., Dong P. (2024). Insights into the plasmonic “hot spots” and efficient hot electron injection induced by Ag nanoparticles in a covalent organic framework for photocatalytic H_2_ evolution. J. Mater. Chem. A.

[52] Yao Y.H., Li J., Zhang H., Tang H.L., Fang L., Niu G.D., Sun X.J., Zhang F.M. (2020). Facile Synthesis of Covalently Connected rGO-COF Hybrid Material by In-Situ Reaction for Enhanced Visible-light Induced Photocatalytic H_2_ Evolution. J. Mater. Chem. A.

[53] Zhang Y.P., Tang H.L., Dong H., Gao M.Y., Li C.C., Sun X.J., Wei J.Z., Qu Y., Li Z.J., Zhang F.M. (2020). Covalent-Organic Framework Based Z-Scheme Heterostructured Noble-Metal-Free Photocatalysts for Visible-Light-Driven Hydrogen Evolution. J. Mater. Chem. A.

[54] Li Y., Wang J., Xu S., Li M., Chen F. (2024). The preparation of 2D TpPa-COF/2D g-C_3_N_4_ heterojunction via in-situ growth for enhanced visible-light photocatalysis. Int. J. Hydrogen Energy.

