# Supplementary material for: Molecular Ir-Based Coordination Compound Grafted onto Covalent Organic Framework for Efficient Photocatalytic H2 Evolution
Source: Materials (Basel). 2025 Apr 19;18(8):1874. doi: 10.3390/ma18081874 (PMC12028946; doi:10.3390/ma18081874)
Supplement: Supplementary file 1 [file materials-18-01874-s001.zip › materials-3572986-supplementary.pdf]

Molecular Ir-Based Coordination Compound Grafted onto Covalent Organic Framework for Efficient Photocatalytic H_2 Evolution

Chao Wu [1], Haoyan Zhang [2], Xuan Zheng [3], Jing Ding [4], Yuanyuan Li [1,*], Feiyong Chen [1,*] and Zhengfeng Zhao [2]

1 Resources and Environment Innovation Institute, Shandong Jianzhu University, Jinan 250101, China; 13127158129@163.com
2 School of Chemistry and Chemical Engineering, Qilu University of Technology (Shandong Academy of Sciences), Jinan 250353, China; y2463809770@163.com (H.Z.); zzf@qlu.edu.cn (Z.Z.)
3 Business School, Shandong Jianzhu University, Jinan 250101, China; 15688496290@163.com
4 School of Energy and Machinery, Dezhou University, Dezhou 253023, China; dingjing@dzu.edu.cn
* Correspondence: yuanyuanli0022@163.com (Y.L.); chenfeiyong@sdjzu.edu.cn (F.C.)

The formula of apparent quantum yield (AQY).

$$N_P = \frac{Pt\lambda}{hc}$$

$$AQY = \frac{The\ number\ of\ reacted\ electrons}{The\ number\ of\ incident\ photons}$$

$$= \frac{2\times R_{H_2}\times N_A}{N_P}$$

N_P: the number of incident photons

P: light intensity (W)

t: time (s)

λ: wavelenghs (m)

EQE: external quantum efficiency

R_{H_2}: H_2 Evolved (mol)

N_A: Avogadro constant

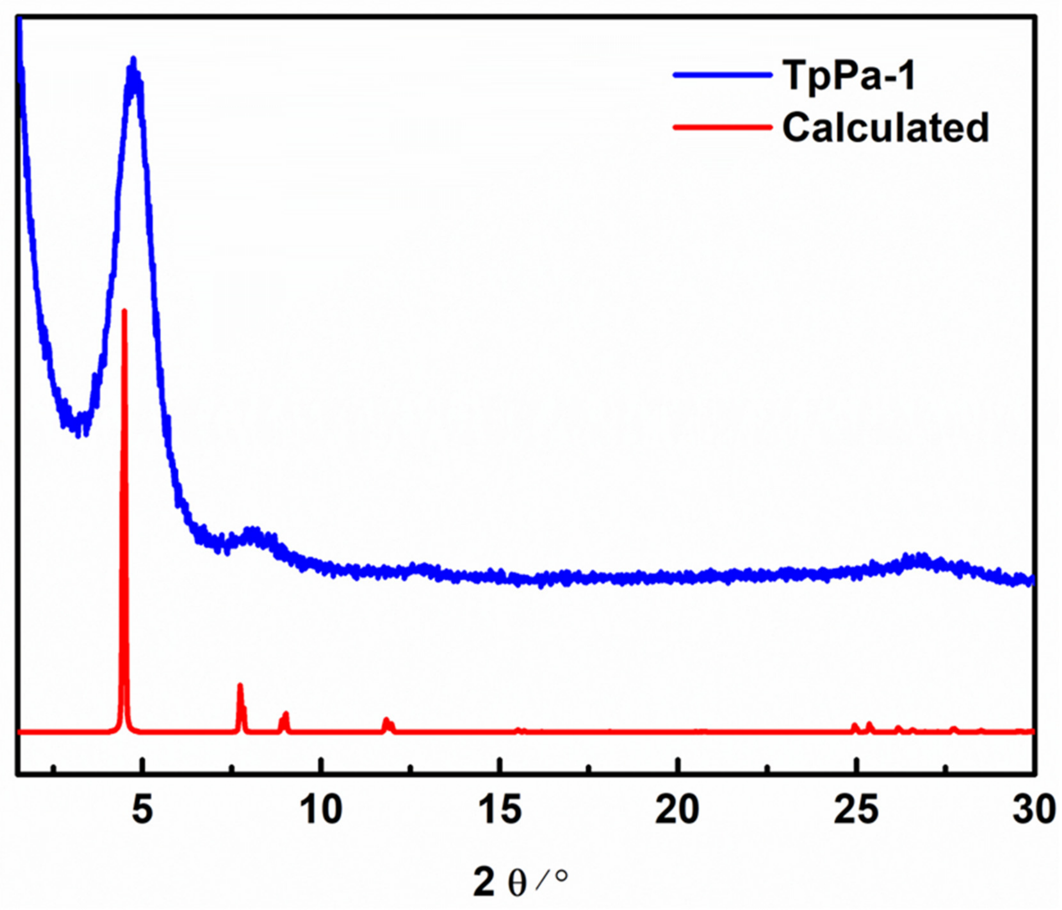

Figure S1. Powder X-ray diffraction (PXRD) analysis of TpPa-COFs.

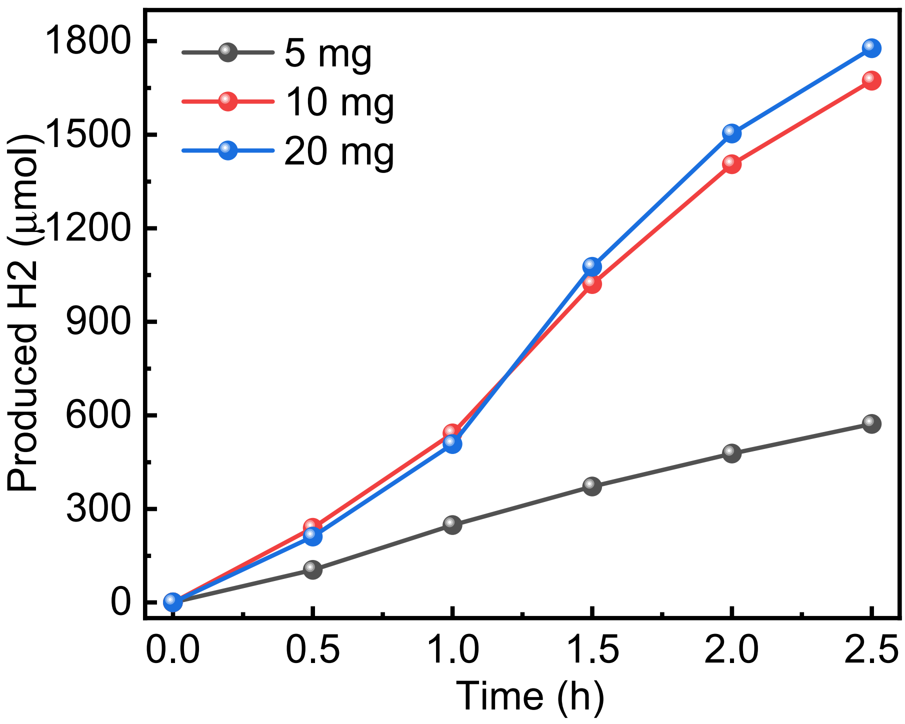

Figure S2. Photocatalytic hydrogen evolution performance of different amounts of catalyst under visible light irradiation.

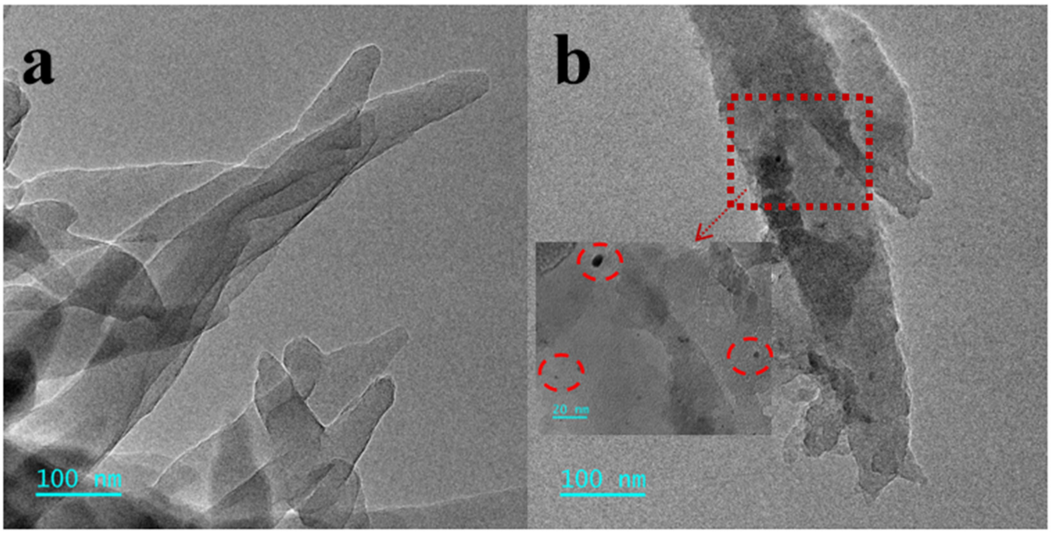

Figure S3. TEM images after the reaction: (a) TpPa-COF+M1; (b) TpPa-COF+M2.

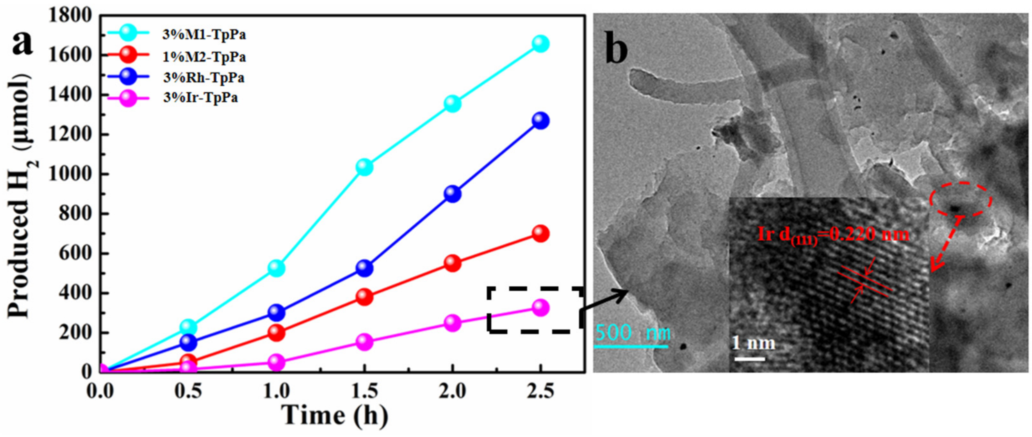

Figure S4. (a):Photocatalytic hydrogen evolution performance of TpPa-COFs with different kinds of molecules ligands under visible light irradiation. 3% Ru-TpPa means that $RhCl_2$ with 3% mass percentage deposited onto TpPa COFs by photodeposition. 3% Ir-TpPa means that $C_{20}H_{30}C_{14}Ir_2$ ((Pentamethylcyclopentadienyl)iridium(III) chloride dimer) with 3% mass percentage deposited onto TpPa COFs by photodeposition. (b) The TEM and HRTEM images of 3% Ir-TpPa after Photocatalytic hydrogen evolution test.

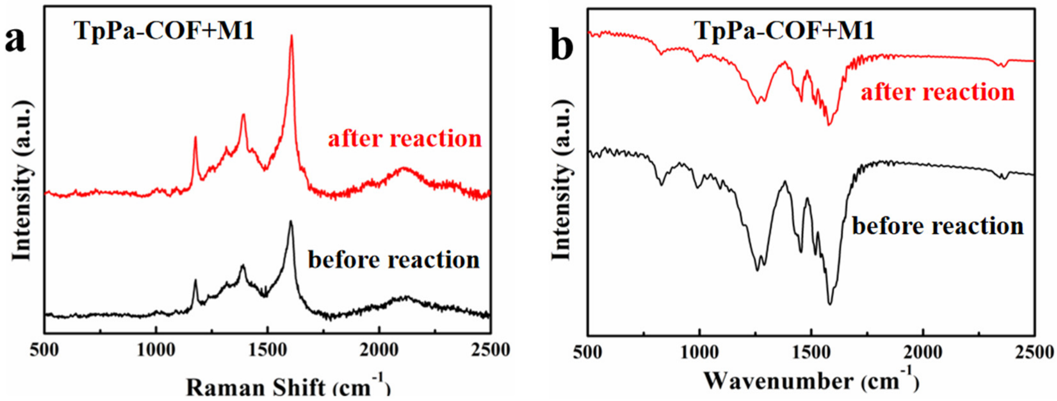

Figure S5. The absorption peaks of TpPa-COFs+M1 before and after photocatalytic hydrogen evolution tests: (a) Raman; (b) FTIR.

Table S1. Ir Mass percentage of different samples tested by ICP.

Samples	Sampling/g	Constant volume/L	Dilution coefficient	Instrument reading	Converted content (mg/L)	Ir Mass percentage（mg/kg）
	A	B	C	D	E	F
1%M1	0.01	25	10	0.2806	7014.69	0.70%
2%M1	0.0098	25	10	0.6506	16596.23	1.66%
3%M1	0.0099	25	10	0.9946	25116.16	2.51%
5%M1	0.011	25	10	1.8636	42354.01	4.24%
1%M2	0.0099	25	10	0.2440	6161.52	0.62%
2%M2	0.01	25	10	0.4439	11096.46	1.11%
3%M1 after reaction	0.009	25	10	0.8459	23497.52	2.35%

Calculation method：E=D*B*C/A，10000mg/kg=1%.

Table S2. Comparison of typical TpPa-COF photocatalysts reported for the hydrogen production efficiency.

Samples	cocatalyst	Irradiation wavelength	Hydrogen production rate (μmol h^{-1})	catalyst dosage (mg)	reference
TpPa-COF+M1	2.5wt%Ir	>420 nm	662	10	(this work)
TiO2-TpPa-1-COF	3wt%Pt	>420 nm	11190	1000	[1]
CdS@TPPA	3wt%Pt	>420 nm	7.956	8	[2]
Ag/TpPa	3wt%Ag	>420 nm	801.4	1000	[3]
rGO(5%)-TpPa-1-COF	3wt%Pt	>420 nm	11980	1000	[4]
Fe_2O_3/TpPa-COF	2wt%Pt	>420 nm	3770	1000	[5]
TpPa-Cl2	3wt%Pt	>420 nm	99260	1000	[6]
TpPa-COF/g-C3N4	3wt%Pt	>420 nm	17600	1000	[7]
TpPaCOF(CH3)2	3wt%Pt	>420 nm	8330	1000	[8]
PtNPs/TpPa	0.5wt%Pt	>420 nm	1100	10	[9]

References

1. Li, C.-C.; Gao, M.-Y.; Sun, X.-J.; Tang, H.-L.; Dong, H.; Zhang, F.-M. Rational combination of covalent-organic framework and nano tio_2 by covalent bonds to realize dramatically enhanced photocatalytic activity. Applied Catalysis B: Environmental 2020, 266, 118586.

2. Chen Y., Yang J.; Gao Y-C., et.al. On-Surface Bottom-Up Construction of COF Nanoshells towards Photocatalytic H_2 Production. Research 2021,12.

3. Zhang LH.;Lu X.; Sun JQ.; et.al. Insights into the plasmonic "hot spots" and efficient hot electron injection induced by Ag nanoparticles in a covalent organic framework for photocatalytic H_2 evolution.Journal of Materials Chemistry A 2024, 12, 5392–5405.

4. Yao YH.; Li J.;,Zhang H.; et.al. Facile Synthesis of Covalently Connected rGO-COF Hybrid Material by In-situ Reaction for Enhanced Visible-light Induced Photocatalytic H_2 Evolution. Journal of Materials Chemistry A 2020.

5. Zhang YP.; Hong-Liang Tang LH.; Dong H.; et. al. Covalent-Organic Framework

Based Z-Scheme Heterostructured Noble-Metal-Free Photocatalysts for Visible-Light-Driven Hydrogen Evolution. Journal of Materials Chemistry A 2020.
6. Jiang SD.;Niu HY.; Sun Q.; et.al. Significant improvement of photocatalytic hydrogen evolution performance in covalent organic frameworks: substituent fine-tuning. Journal of Materials Chemistry A 2024, 12, 11416–11423.
7. Li, YY .; Wang, J. ; Xu, SS.; et.al. The preparation of 2D TpPa-COF/2D g-C_3N_4 heterojunction via in-situ growth for enhanced visible-light photocatalysis. International Journal of Hydrogen Energy 2024, 60,1433-1441.
8. Sheng JL,; Dong H, Meng XB,;et. al. Effect of Different Functional Groups on Photocatalytic Hydrogen Evolution in Covalent-Organic Frameworks. ChemCatChem 2019, 11, 2313–2319.
9. Zhao ZF.; Chen WQ.; Zhang GF.; et. al. Interface molecular wires induce electron transfer from COFs to Pt for enhanced photocatalytic H_2 evolution. Journal of Materials Chemistry A 2023, 11, 26052–26062.
